# Supplementary material for: A UMLS-based spell checker for natural language processing in vaccine safety
Source: BMC Med Inform Decis Mak. 2007 Feb 12;7:3. doi: 10.1186/1472-6947-7-3 (PMC1805499; doi:10.1186/1472-6947-7-3)
Supplement: Additional file 13 — RAP application directory. Sets up directory for RAP (RDF application for PHP) source code [file 1472-6947-7-3-S13.gz › rap/doc/phpdoc/__filesource/fsource_infModel__infModelInfRule.php.html]

xml version="1.0" encoding="iso-8859-1"?


File Source for InfRule.php


# Source for file InfRule.php

Documentation is available at InfRule.php

```
1. <?php
2. // ----------------------------------------------------------------------------------
3. // Class: InfRule
4. // ----------------------------------------------------------------------------------


8. /**
9. * This class represents a single rule in a RDFS inference model.
10. * It primary constists of a trigger and an entailment.
11. * In the forward-chaining mode (RDFSFModel) a statement is checked,
12. * if it satisfies the trigger. If it does, a new statement is returned.
13. * In the backward-chaining mode (RDFSBModel) a find-query is checked
14. * with the entailment. If this entailment could satify the find-query,
15. * a new find-query is returned, that searches for statements that
16. * satisfy the trigger of this rule.
17. *
18. * <BR><BR>History:<UL>
19. * <LI>09-10-2004                : First version of this class.</LI>
20. *
21. * @version  V0.9.1
22. * @author Daniel Westphal <mail at d-westphal dot de>
23. *
24. * @package infModel
25. * @access    public
26. ***/

28. class InfRule
29. {

31. /**
32. * Array, that hold the trigger subject in key ['s'], the trigger
33. * predicate in ['p'], and the trigger object in ['o'].
34. * The array values can be NULL to match anything or be a node that
35. * has to be matched.
36. * @var        array
37. * @access    private
38. */
39. var $trigger;

41. /**
42. * Array, that hold the entailment subject in key ['s'], the
43. * entailment predicate in ['p'], and the entailment object in ['o'].
44. * The array values can be a node that will be inserted in the
45. * returning statement, or '<s>' to insert the subject,'<p>' to insert
46. * the predicate, or '<o>' to insert the object of the checked statement
47. * to this position in the new returned statement.
48. * @var        array
49. * @access    private
50. */
51. var $entailment;


54. /**
55. * Constructor
56. *
57. *
58. * @access    public
59. */
60. function infRule()
61. {
62. //initialising vars
63. $this->trigger=array();
64. $this->entailment=array();
65. }

67. /**
68. * Sets the trigger of this rule
69. * The values can be NULL to match anything or be a node that has to
70. * be matched.
71. * @param    object Node OR NULL    $subject
72. * @param    object Node OR NULL    $predicate
73. * @param    object Node OR NULL    $object
74. * @access    public
75. * @throws    PhpError
76. */
77. function setTrigger ($subject, $predicate, $object)
78. {
79. //throw an error if subject, predicate, or object are neither
80. //node, nor null.
81. if(!is_a($subject,'Node') && $subject != null)
82. trigger_error(RDFAPI_ERROR . '(class: Infrule; method:
83. setTrigger): $subject has to be null or of class Node'
84. , E_USER_ERROR);
85. if(!is_a($predicate,'Node') && $predicate != null)
86. trigger_error(RDFAPI_ERROR . '(class: Infrule; method:
87. setTrigger): $predicate has to be null or of class Node'
88. , E_USER_ERROR);
89. if(!is_a($object,'Node') && $object != null)
90. trigger_error(RDFAPI_ERROR . '(class: Infrule; method:
91. setTrigger): $object has to be null or of class Node'
92. , E_USER_ERROR);

94. //set the trigger
95. $this->trigger['s']=$subject;
96. $this->trigger['p']=$predicate;
97. $this->trigger['o']=$object;
98. }

100. /**
101. * Sets the entailment of this rule
102. * The values can be NULL to match anything or be a node that has to
103. * be matched.
104. * @param    object Node OR NULL    $subject
105. * @param    object Node OR NULL    $predicate
106. * @param    object Node OR NULL    $object
107. * @access    public
108. * @throws    PhpError
109. */
110. function setEntailment($subject,$predicate,$object)
111. {
112. //throw an error if subject, predicate, or object are neither node,
113. //nor <s>,<p>,<o>.
114. if(!is_a($subject,'Node') && !ereg('<[spo]>', $subject))
115. trigger_error(RDFAPI_ERROR . '(class: Infrule; method:
116. setEntailment): $subject has to be <s>,<p>,or <o> or of class Node'
117. , E_USER_ERROR);
118. if(!is_a($predicate,'Node') && !ereg('<[spo]>', $predicate))
119. trigger_error(RDFAPI_ERROR . '(class: Infrule; method:
120. setEntailment): $predicate has to be <s>,<p>,or <o> or of class Node'
121. , E_USER_ERROR);
122. if(!is_a($object,'Node') && !ereg('<[spo]>', $object))
123. trigger_error(RDFAPI_ERROR . '(class: Infrule; method:
124. setEntailment): $object has to be <s>,<p>,or <o> or of class Node'
125. , E_USER_ERROR);

127. $this->entailment['s']=$subject;
128. $this->entailment['p']=$predicate;
129. $this->entailment['o']=$object;
130. }

132. /**
133. * Checks, if the statement satisfies the trigger
134. * @param    object Statement
135. * @return     boolean
136. * @access    public
137. * @throws    PhpError
138. */
139. function checkTrigger(& $statement)
140. {
141. //is true, if the trigger is null to match anything
142. //or equals the statement's subject
143. $shouldFireS =     $this->trigger['s'] ==  null ||
144. $this->trigger['s']->equals($statement->getSubject());

146. //is true, if the trigger is null to match anything
147. //or equals the statement's predicate
148. $shouldFireP =     $this->trigger['p'] ==  null ||
149. $this->trigger['p']->equals($statement->getPredicate());

151. //is true, if the trigger is null to match anything
152. //or equals the statement's object
153. $shouldFireO =     $this->trigger['o'] ==  null ||
154. $this->trigger['o']->equals($statement->getObject());

156. //returns true, if ALL are true
157. return $shouldFireS && $shouldFireP && $shouldFireO;
158. }

160. /**
161. * Checks, if this rule could entail a statement that matches
162. * a find of $subject,$predicate,$object
163. * @param    object Statement
164. * @return     boolean
165. * @access    public
166. * @throws    PhpError
167. */
168. function checkEntailment ($subject, $predicate, $object)
169. {
170. //true, if $subject is null, the entailment's subject matches
171. //anything, or the $subject equals the entailment-subject.
172. $matchesS=    $subject ==  null ||
173. !is_a($this->entailment['s'],'Node') ||
174. $this->entailment['s']->equals($subject);

176. //true, if $predicate is null, the entailment's predicate matches
177. //anything, or the $predicate equals the entailment-predicate.
178. $matchesP=    $predicate ==  null ||
179. !is_a($this->entailment['p'],'Node') ||
180. $this->entailment['p']->equals($predicate);

182. //true, if $object is null, the entailment's object matches
183. //anything, or the $object equals the entailment-object.
184. $matchesO=    $object ==  null ||
185. !is_a($this->entailment['o'],'Node') ||
186. $this->entailment['o']->equals($object);

188. //returns true, if ALL are true
189. return $matchesS && $matchesP && $matchesO;
190. }

192. /**
193. * Returns a infered InfStatement by evaluating the statement with
194. * the entailment rule.
195. * @param    object Statement
196. * @return     object InfStatement
197. * @access    public
198. * @throws    PhpError
199. */
200. function entail(& $statement)
201. {
202. //if the entailment's subject is <s>,<p>,or <o>, put the statements
203. //subject,predicate,or object into the subject of the
204. //entailed statement. If the entailment's subject is a node,
205. //add that node to the statement.
206. switch ($this->entailment['s'])
207. {
208. case '<s>':
209. $entailedSubject=$statement->getSubject();
210. break;
211. case '<p>':
212. $entailedSubject=$statement->getPredicate();
213. break;
214. case '<o>':
215. $entailedSubject=$statement->getObject();
216. break;
217. default:
218. $entailedSubject=$this->entailment['s'];
219. };

221. //if the entailment's predicate is <s>,<p>,or <o>, put the
222. //statements subject,predicate,or object into the predicate of
223. //the entailed statement. If the entailment's predicate is a node,
224. //add that node to the statement.
225. switch ($this->entailment['p'])
226. {
227. case '<s>':
228. $entailedPredicate=$statement->getSubject();
229. break;
230. case '<p>':
231. $entailedPredicate=$statement->getPredicate();
232. break;
233. case '<o>':
234. $entailedPredicate=$statement->getObject();
235. break;
236. default:
237. $entailedPredicate=$this->entailment['p'];
238. };

240. //if the entailment's object is <s>,<p>,or <o>, put the
241. //statements subject,predicate,or object into the object of
242. //the entailed statement. If the entailment's object is a node,
243. //add that node to the statement.
244. switch ($this->entailment['o'])
245. {
246. case '<s>':
247. $entailedObject=$statement->getSubject();
248. break;
249. case '<p>':
250. $entailedObject=$statement->getPredicate();
251. break;
252. case '<o>':
253. $entailedObject=$statement->getObject();
254. break;
255. default:
256. $entailedObject=$this->entailment['o'];
257. };

259. //return the infered statement
260. return (new InfStatement($entailedSubject,$entailedPredicate,$entailedObject));
261. }

263. /**
264. * Returns a find-query that matches statements, whose entailed
265. * statements would match the supplied find query.
266. * @param    Node OR null $subject
267. * @param    Node OR null $predicate
268. * @param    Node OR null $object
269. * @return     array
270. * @access    public
271. * @throws    PhpError
272. */
273. function getModifiedFind( $subject, $predicate, $object)
274. {
275. $findSubject=$this->trigger['s'];
276. $findPredicate=$this->trigger['p'];
277. $findObject=$this->trigger['o'];

279. switch ($this->entailment['s'])
280. {
281. case '<s>':
282. $findSubject=$subject;
283. break;
284. case '<p>':
285. $findPredicate=$subject;
286. break;
287. case '<o>':
288. $findObject=$subject;
289. break;
290. };

292. switch ($this->entailment['p'])
293. {
294. case '<s>':
295. $findSubject=$predicate;
296. break;
297. case '<p>':
298. $findPredicate=$predicate;
299. break;
300. case '<o>':
301. $findObject=$predicate;
302. break;
303. };

305. switch ($this->entailment['o'])
306. {
307. case '<s>':
308. $findSubject=$object;
309. break;
310. case '<p>':
311. $findPredicate=$object;
312. break;
313. case '<o>':
314. $findObject=$object;
315. break;
316. };

318. return array('s' => $findSubject,
319. 'p' => $findPredicate,
320. 'o' => $findObject );
321. }

323. function getTrigger()
324. {
325. return array     (    's' => $this->trigger['s'],
326. 'p' => $this->trigger['p'],
327. 'o' => $this->trigger['o'],
328. );
329. }

331. function getEntailment()
332. {
333. return array     (    's' => $this->entailment['s'],
334. 'p' => $this->entailment['p'],
335. 'o' => $this->entailment['o'],
336. );
337. }
338. }
339. ?>
```

Documentation generated on Thu, 7 Jul 2005 13:41:44 +0200 by phpDocumentor 1.3.0RC3
